# Flame-Retardant Cycloaliphatic Epoxy Systems with High Dielectric Performance for Electronic Packaging Materials

**DOI:** 10.3390/ijms24032301

**Published:** 2023-01-24

**Authors:** Xiao-Wei Jia, Wen-Long Mu, Zhu-Bao Shao, Ying-Jun Xu

**Affiliations:** Institute of Functional Textiles and Advanced Materials, National Engineering Research Center for Advanced Fire-Safety Materials D & A (Shandong), College of Textiles & Clothing, Qingdao University, Qingdao 266071, China

**Keywords:** flame retardancy, cycloaliphatic epoxy resin, dielectric performance, thermal stability, chemical degradation

## Abstract

Flame-retardant cycloaliphatic epoxy systems have long been studied; however, the research suffers from slow and unsatisfactory advances. In this work, we synthesized a kind of phosphorus-containing difunctional cycloaliphatic epoxide (called BCEP). Then, triglycidyl isocyanurate (TGIC) was mixed with BCEP to achieve epoxy systems that are rich in phosphorus and nitrogen elements, which were cured with 4-methylhexahydrobenzene anhydride (MeHHPA) to obtain a series of flame-retardant epoxy resins. Curing behaviors, flame retardancy, thermal behaviors, dielectric performance, and the chemical degradation behaviors of the cured epoxy system were investigated. BCEP-TGIC systems showed a high curing activity, and they can be efficiently cured, in which the incorporation of TGIC decreased the curing activity of the resin. As the ratio of BCEP and TGIC was 1:3, the cured resin (BCEP_1_-TGIC_3_) showed a relatively good flame retardancy with a limiting oxygen index value of 25.2%. In the cone calorimeter test, they presented a longer time to ignition and a lower heat release than the commercially available cycloaliphatic epoxy resins (ERL-4221). BCEP-TGIC systems presented good thermal stability, as the addition of TGIC delayed the thermal weight loss of the resin. BCEP_1_-TGIC_3_ had high dielectric performance and outperformed ERL-4221 over a frequency range of 1 HZ to 1 MHz. BCEP_1_-TGIC_3_ could achieve degradation under mild conditions in an alkali methanol/water solution. Benefiting from the advances, BCEP-TGIC systems have potential applications as electronic packaging materials in electrical and electronic fields.

## 1. Introduction

Epoxy resins (EP) show advantages in the ease of operating, low shrinkage upon curing, superior adhesion, outstanding thermal resistance, good electrical insulation, and fine mechanical performance [1,2,3,4]. Thus, the resin has been widely applied in electronic fields, including conductive adhesives, conformal coatings, printed circuit boards, and flip-chip encapsulation, etc. [5,6]. Among them, cycloaliphatic epoxides, featuring in the aliphatic backbone and fully saturated structure, have attracted much attention for years and have become a good choice in high-frequency electronic devices requiring low dielectric constant and dielectric loss [7,8,9,10]. Usually, cycloaliphatic epoxides are synthesized by the peroxidation of aliphatic alkenes, rather than through the condensation of bisphenol A with epichlorohydrin. They are free of chloride and are often cured with anhydrides. As a result, cycloaliphatic epoxy resins outperform the widely used diglycidyl ether of bisphenol A (DGEBA)-type resins in the dielectric performance [11,12]. In addition, cycloaliphatic EP present good weather and UV resistance, owing to the absence of aromatic groups in the molecular structure [13,14]. For this, they can be very durable as indoor and outdoor power equipment in indoor and outdoor conditions; e.g., transformers, HV generators, motors, and switchgear. However, cycloaliphatic epoxy materials are very easy to catch fire and they then burn violently, accompanied by dripping due to their relatively high contents of aliphatic and cycloaliphatic segments [15,16].

Halogen-based flame retardants, including some high-efficiency brominated flame retardants, gradually become estranged from the mainstream of electronic fields, due to the consequent environmental problems, human health issues, and recycling requirements of electric equipment [17,18]. For this, halogen-free flame retardants, especially phosphorus-containing ones, have been valued for their chemical versatility, environmental friendliness, and high flame-retardant activities in both the gaseous and condensed phases [19,20]. In addition, intrinsic flame-retardant epoxy materials are more highly welcomed than other methods and approaches (e.g., additives and coatings) in the field, as they often exhibit high flame retardancy with well-balanced performance and environmental tolerance [21,22]. Using functional epoxy monomers or curing agents, intrinsic flame-retardant resins can be achieved by incorporating flame-retardant elements and groups into the macromolecular chain [23,24]. Nonetheless, concerning cycloaliphatic epoxy systems, some phosphorus-containing epoxy monomers have been prepared in the past few years, while developing the modified anhydrides as the flame-retardant curing agent is rarely reported. Wang et al. [25,26] developed a series of phosphorus-containing cycloaliphatic epoxy monomers, aiming for the recycling of the integrated circuit without damaging the circuit board under heating. Interestingly, the cured resins rapidly decomposed and then completely lost their strength under relatively low temperatures, lying within the desired temperature range for reworking operation. However, the obtained resins showed unsatisfactory flame retardancy and had a relatively low limiting oxygen index value (not higher than 23.9%). At present, it is still a big challenge for producing halogen-free flame-retardant cycloaliphatic epoxy materials.

Aiming for obtaining flame-retardant epoxy systems with a good insulation performance for electronic packaging materials, in this work, we synthesized a kind of phosphorus-containing difunctional cycloaliphatic epoxide (called BCEP) through the epoxidation of phosphorus-containing olefin (BCP), using diphenyl phosphoryl chloride and 3-cyclohexene-1-methanol as the precursor. Then, triglycidyl isocyanurate (TGIC) was further mixed with BCEP in different proportions to achieve epoxy systems that are rich in phosphorus and nitrogen elements, which was then cured with 4-methylhexahydrobenzene anhydride (MeHHPA) to obtain a series of intrinsic flame-retardant epoxy materials. In the system, TGIC was considered to improve the flame retardancy of the resin through the so-called phosphorus-nitrogen synergism effect. The curing kinetics of the epoxy system was investigated using the non-isothermal procedure of differential scanning calorimetry (DSC) at different heating rates. Furthermore, the flame retardancy and burning behaviors of the cured resin were measured using the limiting oxygen index (LOI) and cone calorimeter test (CCT). The thermal stability of the cured resin was studied via thermogravimetric analysis (TG). The frequency dependence of the dielectric constant, and dielectric loss for the materials was determined using a broadband dielectric spectrometer. In addition, the chemical degradation behavior of the cured resin under mild conditions was studied. In addition, a kind of commercially available cycloaliphatic epoxides (named ERL-4221) cured in the same condition was applied as the reference sample to compare with the obtained BCEP-TGIC resins.

## 2. Results and Discussion

### 2.1. Characterization of BCP and BCEP

**Figure 1:**
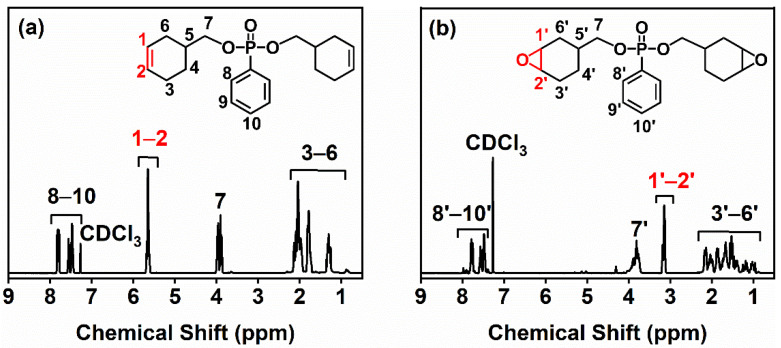
^1^H NMR spectra of BCP (**a**) and BCEP (**b**).

The chemical structures of BCP and BCEP were determined via ^1^H nuclear magnetic resonance (^1^H NMR). The ^1^H NMR spectra are as shown in Figure 1. It can be found that all signals of BEP and BCEP were identified and annotated; e.g., the signals ranging over 7.5–8.0 ppm, 4.5–5.0 ppm, and 0.8–2.5 ppm are severally attributed to the benzene and cycloaliphatic groups (−CH− or −CH_2_−) [27,28]. Especially, the changes in the chemical shift assigned to the olefin and epoxy groups were noted and highlighted. In Figure 1a, a strong signal of the BCP marked as 1 and 2, corresponding to the carbon-carbon double bond emerges at 5.60 ppm, which completely disappears after the epoxidation in the spectrum of the BCEP, as presented in Figure 1b. Instead, a signal labeled as 1’ and 2’ at around 3.15 ppm that is attributed to the epoxy appears for the BCEP. Thus, it confirmed that the carbon-carbon double bonds in the BCP have been completely converted to epoxy groups.

### 2.2. Curing Behaviors of the BCEP-TGIC System

**Figure 2:**
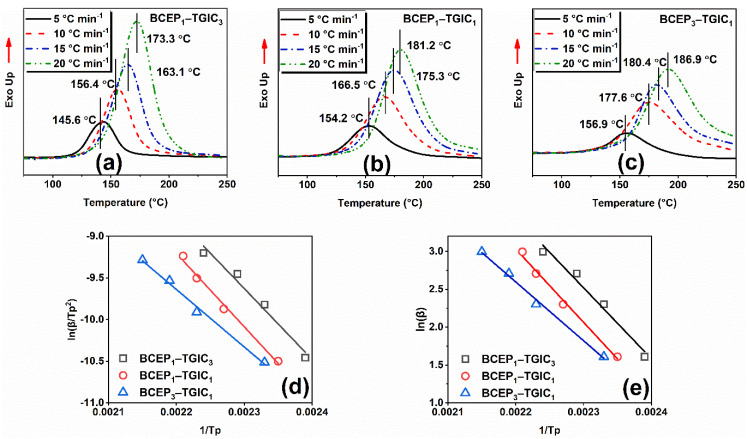
Non-isothermal DSC curves of BCEP-TGIC (**a**–**c**) with different heating rates, linear fitting plots of ln(β/T_p_^2^) versus 1/T_p_ according to the Kissinger equation (**d**), and ln(β) versus 1/T_p_ according to the Ozawa equation (**e**) for BCEP-TGIC.

The curing kinetics of the epoxy system were studied using the DSC non-isothermal procedure at heating rates of 5, 10, 15, and 20 °C min^−1^. Figure 2a–c show the curves of heat flow values versus temperatures of BCEP_1_-TGIC_3_, BCEP_1_-TGIC_1_, and BCEP_3_-TGIC_1_, all of which present a single peak with a different exothermic peak temperature (T_p_). This indicates that BCEP-TGIC systems show a relatively high curing activity and they can be efficiently cured under moderate conditions. Then, curing kinetic parameters are listed in Table 1, including activation energy (*Eα*) and the Arrhenius pre-exponential factor (*A*), evaluated using the Kissinger equation and the Ozawa equation [29,30], where *β* represents the heating rate of the non-isothermal scan. According to the equations, the *E_α_* and *A* values can be determined from the slope and intercept of the linear fitting plots of ln(β/T_p_^2^) versus 1/T_p_ and ln(*β*) versus 1/T_p_, as presented in Figure 2d,e. With the content of TGIC in BCEP-TGIC increasing, *E_α_* calculated using the Kissinger equation (*E_αk_*) and the Ozawa equation (*E_αo_*) of the system increases; e.g., the *E_αk_* and E_αo_ of BCEP_1_-TGIC_3_, BCEP_1_-TGIC_1_, and BCEP_3_-TGIC_1_ are 70.6 and 74.1 kJ mol^−1^, 73.2 and 76.5 kJ mol^−1^, and 57.4 and 61.4 kJ mol^−1^, respectively. Therefore, the incorporation of TGIC can lower the curing activity of the epoxy system. This phenomenon can be explained as follows. Usually, cycloaliphatic epoxides have a higher reactivity to anhydrides than glycidyl ethers, because they tend to ring opening more easily at high temperatures than the latter. In addition, the isocyanurate of TGIC, having a strong electron-withdrawing ability, decreases the reactivity of the glycidyl ether with anhydride curing agents [26,31].

### 2.3. Flame Retardancy and Burning Behaviors of the Cured Epoxy System

We measured the flame retardancy of the cured epoxy system using the LOI and UL-94 test, and the obtained data are compared in Table 2. It can be observed that the ERL-4221 resins cured with MeHHPA only have a low LOI value to 18.0%, indicating that they are highly flammable and that it is necessary to develop a flame-retardant cycloaliphatic epoxy system. In contrast, the BCEP cured in the same conditions presents a higher LOI of 22.4%. Interestingly, when incorporating TGIC into the system, all of the cured samples show a relatively high LOI value, although the cured TGIC resins underperform in flame retardancy (having an LOI value of 23.2%). Among them, the cured BCEP_1_-TGIC_3_, which has a calculated phosphorus content of 0.86 wt.% and a nitrogen content of 1.44 wt.%, achieves the highest LOI value of 25.2%. Thus, it can be confirmed that mixing nitrogen-enriched TGIC with phosphorus-containing BCEP is a good choice for preparing a flame-retardant epoxy system. In addition, above results suggest that the phosphorus-nitrogen synergism contributes to the improved flame retardancy of the resin. However, all of the samples achieve no ratings in the UL-94 test, although BCEP_3_-TGIC_1_ shows a relatively low flame spread rate during the test and leaves some dense residues after the test. 

**Figure 3:**
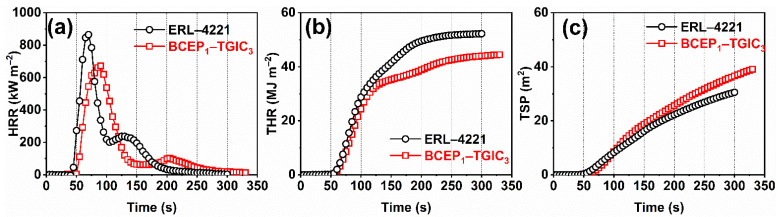
HRR (**a**), THR (**b**), and TSP (**c**) curves of the cured ERL-4221 and BCEP_1_-TGIC_3_ recorded by the cone calorimeter.

Burning behaviors of the cured BCEP_1_-TGIC_3_ are further investigated using the CCT, where the cured ERL-4221 resins are employed as the contrast sample for comparison. Considering that the chemical structures of the two resins are quite different, we just qualitatively evaluate the flame retardancy of the cured resins according to the results of the CCT. Figure 3 depicts the curves of heat release rate (HRR), total heat release rate (THR), and total smoke release rate (TSP). Some important parameters obtained from the test are listed in Table 3, including the time to ignition (TTI), peak of HRR (PHRR), time to PHRR (TTPHRR), THR, TSP, fire growth rate (FIGRA = max value of HRR(t)/t), and char residues. BCEP_1_-TGIC_3_ present a little longer TTI and TTPHRR than ERL-4221, indicating that they perform well in delaying ignition and fire growth during the initial stage of the test, due to the introduction of flame-retardant groups. Usually, PHRR is considered as the most important parameter to evaluate the fire-safety performance of the material [32,33]. It is noted that BCEP_1_-TGIC_3_ achieves a lower PHRR and THR than the reference sample; in addition, it shows a relatively low FIGRA value of 7.5 kW·m^−2^ s^−1^ (FIGRA of ERL-4221 is 12.3 kW·m^−2^ s^−1^). This indicates that the flame-retardant epoxy system presents a relatively low heat release rate and fire spread rate under simulated fire conditions [34,35]. In addition, due to the incomplete combustion of the flame-retardant material, the cured BCEP_1_-TGIC_3_ show a higher TSP than the cured ERL-4221 (39.1 m^2^ versus 30.5 m^2^) and it finally achieves more char residues than the contrast sample (0 versus 4.8%). According to the above results, the cured BCEP_1_-TGIC_3_ outperforms the commercially available ERL-4221 in flame retardancy, and thus exhibits the potential for versatile applications in electrical and electronic fields.

### 2.4. Thermal Behaviors of the Cured Epoxy System

**Figure 4:**
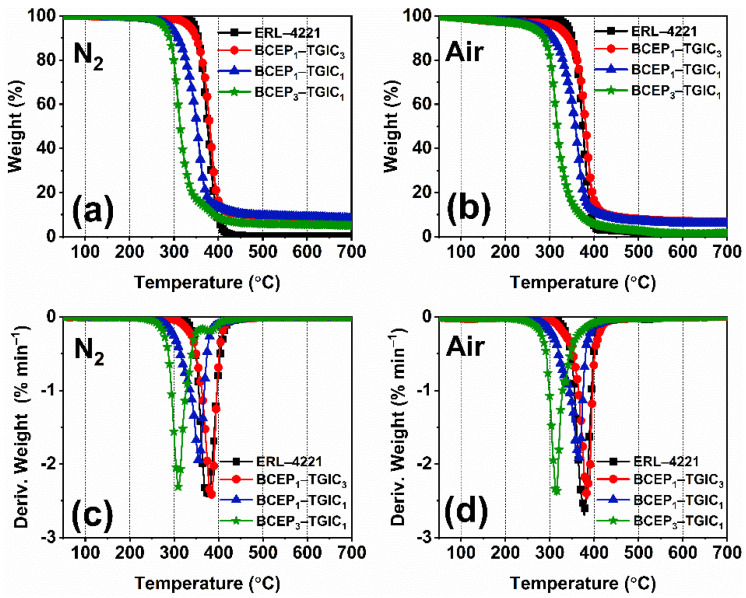
TG (**a**,**b**) and DTG (**c**,**d**) curves of the cured resin under nitrogen (**a**,**c**) and air (**b**,**d**) atmospheres.

TG was conducted to study the thermal stability of the cured epoxy system. The TG and derivative TG (DTG) curves under nitrogen and air atmospheres of the cured ERL-4221 and BCEP-TGIC samples are plotted in Figure 4, and the data collected from the curve are listed in Table 4. Under the nitrogen and air atmospheres, the cured BCEP-TGIC samples present a one-stage thermal weight loss course ranging from 250 °C to 400 °C, which is mainly attributed to the degradation of the cured epoxy networks. When the content of TGIC in the BCEP-TGIC increases, the initial weight-loss temperature (T_i_) and maximum weight-loss temperature (T_max_) of the cured resin improve; e.g., the cured BCEP_1_-TGIC_3_ shows a T_i_ of 318 °C and a T_max_ of 387 °C under the nitrogen atmosphere and presents a T_i_ of 335 °C and a T_max_ of 382 °C under the air atmosphere, indicating a good thermal stability of the flame-retardant epoxy system. Note that with the content of BCEP increasing, the residual weight of the cured resins at 700 °C (W_700_) exhibits a trend of decreasing (lower than 10%). This is because the phosphate bonds of BCEP are very sensitive to temperature, causing the resin to decompose quickly over a narrow temperature range. In addition, the glass transition temperature (T_g_) of the cured epoxy system was determined using DSC and dynamic thermomechanical analysis (DMA), as listed in Table 2. It is known that the crosslinking density greatly influences the thermomechanical behavior of a resin. BCEP-TGIC systems with a higher content of bi-functional BCEP present a lower T_g_, while the cured BCEP_1_-TGIC_3_ shows a relatively high T_g_ of about 200 °C. According to the results, it can be found that BCEP-TGIC systems show good heat resistance, with a relatively high T_i_ and T_g_, even better than those of DGEBA-type resins [36,37], and thus, they can tolerate a high manufacturing and working temperature of electronic and electrical products.

### 2.5. Dielectric Properties of the Cured Epoxy System

**Figure 5:**
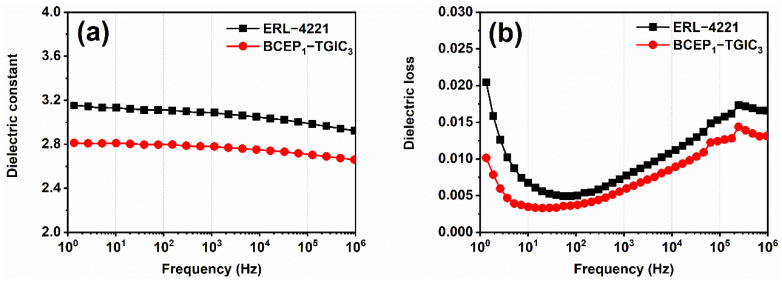
Dielectric constant (**a**) and dielectric loss (**b**) versus the frequency curves of the cured ERL-4221 and BCEP_1_-TGIC_3_.

Low dielectric constant and dissipation energy under high frequency is one of the most important parameters for insulating materials to maintain signal propagation and the safe running of electronic devices [38,39]. Thus, the dielectric performance of the cured epoxy system was studied using a broadband dielectric spectrometer. Dielectric constant (ε) and dielectric loss (tan δ) versus the frequency of the resin are plotted in Figure 5. Over the wide frequency range of 1 HZ-10^6^ Hz (1 MHz), the cured BCEP_1_-TGIC_3_ presents a relatively low ε compared to ERL-4221, which decreases slowly from 2.8 to 2.7, with the frequency increasing. This indicates that BCEP_1_-TGIC_3_ has a low charge storage ability with the presence of electric fields. In addition, the cured BCEP_1_-TGIC_3_ shows the same frequency dependence tendency of tan δ as the cured ERL-4221, whereas the tan δ value of the cured BCEP_1_-TGIC_3_ is lower than the cured ERL-4221 from 1 HZ to 1 MHz. This suggests that the cured BCEP_1_-TGIC_3_ tends to consume very little electric energy to heat in an alternating electric field [40,41]. The above results suggest that the cured BCEP_1_-TGIC_3_ has good insulating properties, and thus, shows that it can meet the high dielectric requirements for electric/electronic applications. We demonstrate that the low content of polar groups in the BCEP_1_-TGIC_3_, high crosslinking density with the large steric hindrance of groups (e.g., isocyanurate and cycloaliphatic groups) in the network, and fine structure symmetry of both BCEP and TGIC can be the reason for why the cured BCEP_1_-TGIC_3_ achieves a good dielectric performance.

### 2.6. Chemical Degradation Behaviors of the Cured Epoxy System

**Figure 6:**
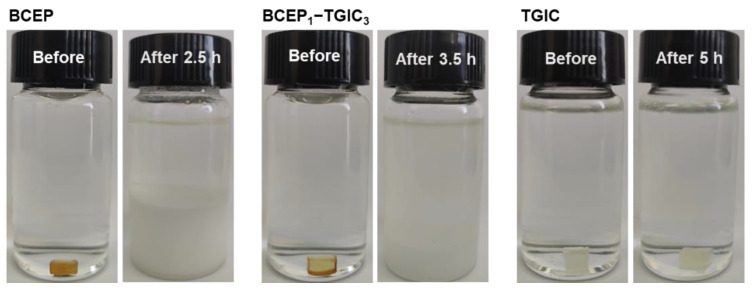
Photographs of the cured BCEP, BCEP_1_-TGIC_3_, and TGIC before and after chemical degradation in a methanol/water solution of NaOH under 50 °C.

Facing the increasing demand for the disposal, recycling, and reuse of waste electronic devices [42,43], epoxy systems that can degrade in a moderate condition are strongly desired as electronic packaging materials to meet environmental and cost-saving requirements. It is noted that phosphate bonds have been introduced into epoxy networks as active moieties to achieve chemical/thermal degradation characteristics [44,45]. We studied the chemical degradation behaviors of the cured epoxy systems to further understand the cured network of BCEP-TGIC systems. Considering the degradation mode of the epoxy system, we applied an alkali methanol/water solution to investigate the chemical degradation behavior of the cured BCEP-TGIC systems. Herein, the cured resins were added to a methanol/water solution of NaOH (0.5 mol L^−1^) under a temperature of 50 °C. Figure 6 presents the photo of the cured BCEP, BCEP_1_-TGIC_3_, and TGIC before and after the treatment. After 2.5 h, the cured BCEP can quickly degrade and result in a white precipitate at the bottom of the bottle. In contrast, the cured BCEP_1_-TGIC_3_ breaks up into white particles in 3.5 h, while the cured TGIC keeps stable for 5 h under the same conditions, indicating that the BCEP endows the epoxy system with degradation capacity. We believe the degradation of the cured BCEP and BCEP_1_-TGIC_3_ can be due to the hydrolysis and alcoholysis of the phosphate bond in NaOH methanol/water solutions.

## 3. Materials and Methods

### 3.1. Materials

Phenylphosphonic dichloride (AR, 98%), 3-cyclohexene-1-methanol (AR, 98%), triethylamine (AR, 99%), 3-chloroperbenzoic acid (m-CPBA) (AR, 85%), dichloromethane (CH_2_Cl_2_) (AR, 99.5%), anhydrous magnesium sulfate (MgSO_4_) (AR, 99%), and sodium bicarbonate (NaHCO_3_) and TGIC (AR, 98%), were provided by Macklin Chemical Reagent Co., Ltd., Shanghai, China. 3, 4-Epoxycyclohexene methyl 3, 4-epoxycyclohexenate (ERL-4221) (AR, 97%) was obtained from Aladdin Biochemical Technology Co., Ltd., Shanghai, China. MeHHPA (AR, 98%) was supplied by Chengdu West Asia Chemical Co., Ltd., Chengdu, China. All raw materials and chemical reagents were used as received without further purification.

### 3.2. Synthesis of BCP and BCEP

Under a nitrogen atmosphere, 62.5g 3-cyclohexene-1-methanol (0.56 mol) and 38.2 mL triethylamine (0.26 mol) were added into a flask and dissolved in 100 mL CH_2_Cl_2_. Then, 36.26 g phenylphosphonic dichloride (0.19 mol) was slowly added to the solution in an ice bath. After stirring overnight at room temperature, the obtained mixture was washed with excessive water several times. Then, an organic solution was achieved using the separating funnel and dried over anhydrous MgSO_4_. After filtration, the solvent and excess cyclohex-3-enyl-1-methanol were removed via distillation under reduced pressure, and finally, bis(cyclohex-3-enylmethyl)phenyl phosphate (BCP) was obtained as a brown liquid with a yield of 96%. 

**Scheme 1:**
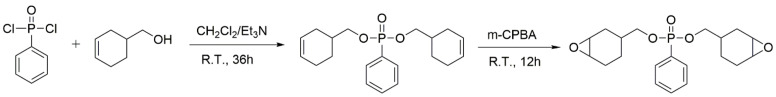
Synthesis route of BCP and BCEP.

In a flask cooled in an ice bath and equipped with a mechanical stirring, 34.6 g BCP (0.10 mol), 41.3 g m-CPBA (0.24 mol), 20.2 g NaHCO_3_ (0.24 mol), and 300 mL CH_2_Cl_2_ were added, and then the mixture was violently stirred for 12 h under a nitrogen atmosphere. After that, the system was quenched using a sodium sulfite solution, and further washed with a NaHCO_3_ solution and deionized water. Then, the organic phase was collected, dried with anhydrous MgSO_4_, and subsequently loaded onto silica gel column chromatography. Finally, the solvent was removed using a rotary evaporator, and 22.5 g of bis((7-oxabicyclo[4.1.0]heptan-3-yl)methyl) phenylphosphonate (BCEP) (yield 65%) was obtained as a yellow liquid with a viscosity of 30 Pa·s at 25 °C. Scheme 1 introduces the full synthesis route of BCEP.

### 3.3. Curing of the Cycloaliphatic Epoxy System

**Scheme 2:**
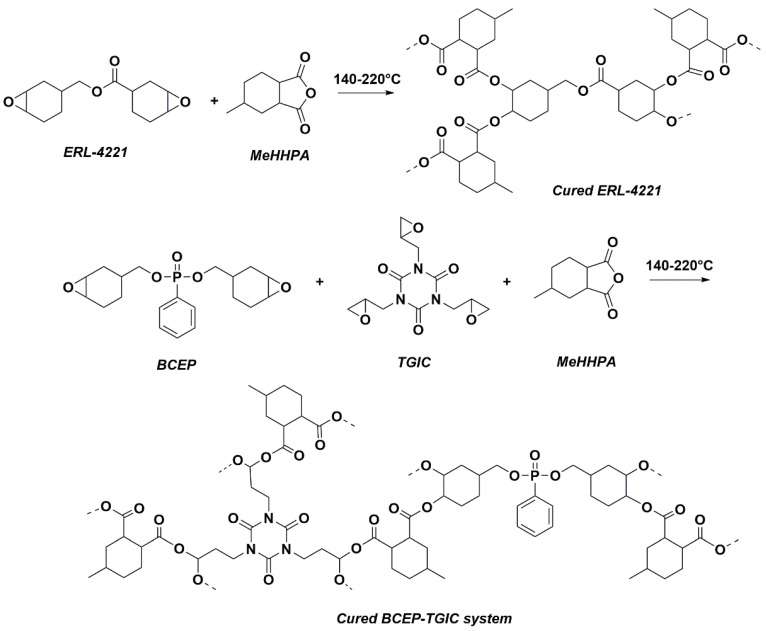
Curing routes of ERL-4221 and BCEP-TGIC MeHHPA-cured networks.

TGIC was added into BCEP, and then the mixture was stirred at 90 °C to achieve a homogeneous solution without any precipitation. Then, a stoichiometric amount of MeHHPA was incorporated into the epoxy systems. After degassing under vacuum at 60 °C, the resin was poured into a pre-heated Teflon mold and then severally cured at 140 °C, 180 °C, and 220 °C for 2 h. After that, homogeneous cured resins were obtained finally. In addition, commercially available ERL-4221 resins were cured with MeHHPA in the same condition for comparison. Formulations of the above epoxy samples are shown in Table 2. Note that the sample was named BCEP_m_-TGIC_n_, in which the mass ratio of BCEP:TGIC was m:n. The reaction processes of ERL-4221 and BCEP-TGIC cured with MeHHPA are presented in Scheme 2.

### 3.4. Characterization

^1^H NMR of BCP and BCEP were recorded on an AVANCE III HD spectrometer (Bruker, Germany) using CDCl_3_ as the solvent. DSC was performed on a TA2500 apparatus (TA, New Castle, Delaware, USA) to study the curing kinetics of the resin under heating rates of 5, 10, 15, and 20 °C min^−1^. The flame retardancy of the cured samples was evaluated via the limiting oxygen index test (LOI) using a sample with a dimension of 130 × 6.5 × 3.2 mm^3^, and the UL-94 test using a specimen with 130 × 13 ×3.2 mm^3^. CCT was conducted on an FTT cone calorimeter (Fire Testing Technology, East Grinstead, West Sussex, UK) according to the ISO 5660 standard, in which the specimen dimension was 100 × 100 × 1.6 mm^3^ and the heat reflux was 35 kW m^−2^. TG analysis was performed by using a TA5500 instrument (TA, New Castle, Delaware, USA) under an air or nitrogen flow of 25 mL min^−1^ with a heating rate of 10 °C min^−1^. DSC analysis was carried out on a TA2500 apparatus (TA, New Castle, DE, USA) at a heating rate of 10 °C min^−1^, and DMA was performed on an RSA G2 instrument (TA, New Castle, DE, USA) under a three-point bending model at a heating rate of 5 °C min^−1^ to determine the T_g_ of the cured sample. The dielectric performance of the sample was studied using a GmbH Concept 80 broadband dielectric spectrometer (Novocontrol, Germany) at room temperature, using a disk sample with a diameter of 35 mm and a thickness of 2 mm.

## 4. Conclusions

We synthesized a kind of difunctional cycloaliphatic epoxide (BCEP), and then developed cycloaliphatic epoxy systems (BCEP-TGIC) by mixing phosphorus-containing BCEP with nitrogen-containing TGIC. It was found via the DSC non-isothermal procedure that the BCEP-TGIC systems showed a high curing activity and they could be efficiently cured, although the incorporation of TGIC lowered the curing activity of the resin. Among the systems, the cured BCEP_1_-TGIC_3_ showed relatively good flame retardancy, with an LOI value of 25.2%. They achieved a longer TTI and a lower heat release in the CCT, compared to the commercially available ERL-4221. TG tests confirmed that the cured BCEP-TGIC systems presented good thermal stability, in which the T_i_ and T_max_ of the cured resin improved, with the content of TGIC increasing. The cured BCEP_1_-TGIC_3_ had a high dielectric performance, with ε and tan δ that were even lower than the cured ERL-4221 over a wide frequency range of 1 HZ to 1 MHz. The cured BCEP_1_-TGIC_3_ showed good chemical degradation behaviors in an alkali methanol/water solution, in which BCEP endowed the resin with a degradation capacity. Using the advantage of good flame retardancy, high thermal stability, superior electrical insulation, and ease of degradation in mild conditions, BCEP-TGIC systems show great potential in the electrical and electronic fields as electronic packaging materials.

## Figures and Tables

**Table 1:** Curing kinetic parameters obtained from non-isothermal DSC scans.

Curing System	*β* (℃ min^−1^)	T_p_ (℃)	*E_αk_* (kJ mol^−1^)	lnA (s^−1^)	n	*E_αo_* (kJ mol^−1^)
BCEP_1_-TGIC_3_	5	144.0	70.6	12.1	0.95	74.1
	10	156.1				
	15	164.6				
	20	172.5				
BCEP_1_-TGIC_1_	5	153.9	73.2	12.3	0.91	76.5
	10	167.3				
	15	175.3				
	20	180.2				
BCEP_3_-TGIC_1_	5	156.7	57.4	7.5	0.89	61.4
	10	176.3				
	15	182.2				
	20	191.7				

**Table 2:** Formula and flame retardancy test results of the epoxy system.

Sample	ERL-4221 (g)	BCEP (g)	TGIC (g)	MeHHPA (g)	P ^a^ (wt.%)	N ^b^ (wt.%)	LOI (%)	UL-94
ERL-4221	25.20	/	/	28.56	/	/	18.0	N.R. ^c^
BCEP	/	37.80	/	28.60	4.82	/	22.4	N.R.
BCEP_3_-TGIC_1_	/	11.34	3.78	15.12	3.17	0.59	23.9	N.R.
BCEP_1_-TGIC_1_	/	3.78	3.78	9.41	1.89	1.05	24.2	N.R.
BCEP_1_-TGIC_3_	/	3.78	11.34	22.01	0.86	1.44	25.2	N.R.
TGIC	/	/	29.70	50.40	/	1.75	23.2	N.R.

^a^ calculated phosphorus content; ^b^ calculated nitrogen content; ^c^ no rating.

**Table 3:** CCT data of the cured ERL-4221 and BCEP_1_-TGIC_3_ under a heat flux of 35 kW m^−2^.

Sample	TTI (s)	PHRR (kW·m^−2^)	TTPHRR (s)	FIGRA (kW·m^−2^ s^−1^)	THR (MJ·m^−2^)	TSP (m^2^)	Residues (%)
ERL-4221	39	864	70	12.3	52.2	30.5	0
BCEP_1_-TGIC_3_	43	671	90	7.5	44.1	39.1	4.8

**Table 4:** TG data of the cured resins and their T_g_ acquired from DSC and DMA.

Sample	TG (N_2_)			TG (Air)			DSC	DMA
	T_i_ (℃)	T_max_(℃)	W_700_ (%)	T_i_ (℃)	T_max_ (℃)	W_700_ (%)	T_g_ (℃)	T_g_ (℃)
ERL-4221	347	372	0.1	340	378	0.6	239	240
BCEP_1_-TGIC_3_	335	382	8.3	318	387	6.9	198	200
BCEP_1_-TGIC_1_	291	356	8.1	271	360	5.9	188	/
BCEP_3_-TGIC_1_	278	310	4.9	258	315	1.5	179	/

## Data Availability

The data are available upon request.

## References

[1] Xu B., Liu Y., Wei S., Zhao S., Qian L., Chen Y., Shan H., Zhang Q. (2022). A Phosphorous-Based Bi-Functional Flame Retardant Based on Phosphaphenanthrene and Aluminum Hypophosphite for an Epoxy Thermoset. Int. J. Mol. Sci..

[2] Lu J.-H., Xu Y.-J., Chen L., Chen J.-H., He J.-H., Li Z., Li S.-L., Wang Y.-Z. (2022). Facile fabrication of intrinsically fire-safety epoxy resin cured with phosphorus-containing transition metal complexes for flame retardation, smoke suppression, and latent curing behavior. Chem. Eng. J..

[3] Gao T.-Y., Wang F.-D., Xu Y., Wei C.-X., Zhu S.-E., Yang W., Lu H.-D. (2022). Luteolin-based epoxy resin with exceptional heat resistance, mechanical and flame retardant properties. Chem. Eng. J..

[4] Rao W., Tao J., Yang F., Wu T., Yu C., Zhao H.-B. (2023). Growth of copper organophosphate nanosheets on graphene oxide to improve fire safety and mechanical strength of epoxy resins. Chemosphere.

[5] Yang Y., Xu Y., Ji Y., Wei Y. (2021). Functional epoxy vitrimers and composites. Prog. Mater. Sci..

[6] He L., Chen T., Zhang Y., Hu L., Wang T., Han R., He J.-L., Luo W., Liu Z.-G., Deng J.-N. (2022). Imide-DOPO derivative endows epoxy resin with excellent flame retardancy and fluorescence without losing glass transition temperature. Compos. B Eng..

[7] Zhang X.H., Zhang Z.H., Xia X.N., Zhang Z.S., Xu W.J., Xiong Y.Q. (2007). Synthesis and characterization of a novel cycloaliphatic epoxy resin starting from dicyclopentadiene. Eur. Polym. J..

[8] Yoo M.J., Kim S.H., Park S.D., Lee W.S., Sun J.-W., Choi J.-H., Nahm S. (2010). Investigation of curing kinetics of various cycloaliphatic epoxy resins using dynamic thermal analysis. Eur. Polym. J..

[9] Zhao L.N., Wang Z.G. (2017). Synthesis of silicon-containing cycloaliphatic diepoxide from biomass-based α-terpineol and the decrosslinking behavior of cured network. Polymer.

[10] Barabanova A.I., Lokshin B.V., Kharitonova E.P., Afanasyev E.S., Askadskii A.A., Philippova O.E. (2019). Curing cycloaliphatic epoxy resin with 4-methylhexahydrophthalic anhydride: Catalyzed vs. uncatalyzed reaction. Polymer.

[11] Zhao L.N., Liu Y.D., Wang Z.G., Li J.F., Liu W.S., Chen Z. (2013). Synthesis and degradable property of novel sulfite-containing cycloaliphatic epoxy resins. Polym. Degrad. Stabil..

[12] Lu M., Liu Y., Du X., Zhang S., Chen G., Zhang Q., Yao S., Liang L., Lu M. (2019). Cure Kinetics and Properties of High Performance Cycloaliphatic Epoxy Resins Cured with Anhydride. Ind. Eng. Chem. Res..

[13] Huang X.Y., Zheng Y., Jiang P.K., Yin Y. (2010). Influence of nanoparticle surface treatment on the electrical properties of cycloaliphatic epoxy nanocomposites. IEEE Trans. Dielectr. Electr. Insul..

[14] Sivanesan D., Seo B., Lim C.-S., Kim S., Kim H.-G. (2021). Trifunctional cycloaliphatic epoxy-based thermoset polymers: Synthesis, polymerization, and characterization. Polymer.

[15] Chen Z., Zhao L.N., Wang Z.G. (2014). Flame retardancy effects of phosphorus-containing compounds and cationic photoinitiators on photopolymerized cycloaliphatic epoxy resins. J. Appl. Polym. Sci..

[16] Chao P.J., Li Y.J., Gu X.Y., Han D.D., Jia X.Q., Wang M.Q., Zhou T.F., Wang T. (2015). Novel phosphorus–nitrogen–silicon flame retardants and their application in cycloaliphatic epoxy systems. Polym. Chem..

[17] Wager P.A., Schluep M., Muller E., Gloor R. (2012). RoHS regulated substances in mixed plastics from waste electrical and electronic equipment. Environ. Sci. Technol..

[18] Huo S., Song P., Yu B., Ran S., Chevali V.S., Liu L., Fang Z., Wang H. (2021). Phosphorus-containing flame retardant epoxy thermosets: Recent advances and future perspectives. Prog. Polym. Sci..

[19] Liu C., Li P., Xu Y.-J., Liu Y., Zhu P., Wang Y.-Z. (2022). Epoxy/iron alginate composites with improved fire resistance, smoke suppression and mechanical properties. J. Mater. Sci..

[20] Yu C., Wu T., Yang F., Wang H., Rao W., Zhao H.-B. (2022). Interfacial engineering to construct P-loaded hollow nanohybrids for flame-retardant and high-performance epoxy resins. J. Colloid. Interface Sci..

[21] Wang J., Liu W., Liu H., Wang X., Wu D., Zhang S., Shi S., Liu W., Wu Z. (2022). Cyclotriphosphazene-based epoxy resins with excellent mechanical and flame retardant properties. Polymer.

[22] Kandola B.K., Magnoni F., Ebdon J.R. (2022). Flame retardants for epoxy resins: Application-related challenges and solutions. J. Vinyl Addit. Technol..

[23] Ma C., Qian L., Li J. (2021). Effect of functional groups of magnolol-based cyclic phosphonate on structure and properties of flame retardant epoxy resin. Polym. Degrad. Stabil..

[24] Kamalipour J., Beheshty M.H., Zohuriaan-Mehr M.J. (2022). Novel phosphonated hardeners derived from diamino diphenyl sulfone for epoxy resins: Synthesis and one-pack flame-retardant formulation alongside dicyandiamide. Polym. Degrad. Stabil..

[25] Liu W.S., Wang Z.G., Xiong L., Zhao L.N. (2010). Phosphorus-containing liquid cycloaliphatic epoxy resins for reworkable environment-friendly electronic packaging materials. Polymer.

[26] Chen Z., Zhao L.N., Wang Z.G. (2013). Synthesis of phosphite-type trifunctional cycloaliphatic epoxide and the decrosslinking behavior of its cured network. Polymer.

[27] Wang H.L., Liu J.H., Xu S.P., Shi W.F. (2009). Preparation and film properties of tri(3,4-epoxycyclohexylmethyl) phosphate based cationically UV curing coatings. Prog. Org. Coat..

[28] Liu W., Wang Z. (2011). Silicon-Containing Cycloaliphatic Epoxy Resins with Systematically Varied Functionalities: Synthesis and Structure/Property Relationships. Macromol. Chem. Phys..

[29] Ma C., Qiu S.L., Yu B., Wang J.L., Wang C.M., Zeng W.R., Hu Y. (2017). Economical and environment-friendly synthesis of a novel hyperbranched poly(aminomethylphosphine oxide-amine) as co-curing agent for simultaneous improvement of fire safety, glass transition temperature and toughness of epoxy resins. Chem. Eng. J..

[30] Ma J., Li G., Hua X., Liu N., Liu Z., Zhang F., Yu L., Chen X., Shang L., Ao Y. (2022). Biodegradable epoxy resin from vanillin with excellent flame-retardant and outstanding mechanical properties. Polym. Degrad. Stabil..

[31] Kumar S., Krishnan S., Mohanty S., Nayak S.K. (2018). Synthesis and characterization of petroleum and biobased epoxy resins: A review. Polym. Int..

[32] Schartel B., Wilkie C.A., Camino G. (2016). Recommendations on the scientific approach to polymer flame retardancy: Part 1—Scientific terms and methods. J. Fire Sci..

[33] Kong Q., Sun Y., Zhang C., Guan H., Zhang J., Wang D.-Y., Zhang F. (2019). Ultrathin iron phenyl phosphonate nanosheets with appropriate thermal stability for improving fire safety in epoxy. Compos. Sci. Technol..

[34] Zhang F.-Q., Zhao Y.-Z., Xu Y.-J., Liu Y., Zhu P. (2022). Flame retardation of vinyl ester resins and their glass fiber reinforced composites via liquid DOPO-containing 1-vinylimidazole salts. Compos. B Eng..

[35] Zhou L.-L., Li W.-X., Zhao H.-B., Zhao B. (2022). Comparative Study of M(II)Al (M = Co, Ni) Layered Double Hydroxides for Silicone Foam: Characterization, Flame Retardancy, and Smoke Suppression. Int. J. Mol. Sci..

[36] Xu Y.-J., Chen L., Rao W.-H., Qi M., Guo D.-M., Liao W., Wang Y.-Z. (2018). Latent curing epoxy system with excellent thermal stability, flame retardance and dielectric property. Chem. Eng. J..

[37] Song X., Deng Z.-P., Li C.-B., Song F., Wang X.-L., Chen L., Guo D.-M., Wang Y.-Z. (2022). A bio-based epoxy resin derived from p-hydroxycinnamic acid with high mechanical properties and flame retardancy. Chinese Chem. Lett..

[38] Li C., Fan H., Aziz T., Bittencourt C., Wu L., Wang D.-Y., Dubois P. (2018). Biobased Epoxy Resin with Low Electrical Permissivity and Flame Retardancy: From Environmental Friendly High-Throughput Synthesis to Properties. ACS Sustain. Chem. Eng..

[39] Liu X.-F., Xiao Y.-F., Luo X., Liu B.-W., Guo D.-M., Chen L., Wang Y.-Z. (2022). Flame-Retardant multifunctional epoxy resin with high performances. Chem. Eng. J..

[40] Wang B., Liu L., Huang L., Chi L., Liang G., Yuan L., Gu A. (2015). Fabrication and origin of high-k carbon nanotube/epoxy composites with low dielectric loss through layer-by-layer casting technique. Carbon.

[41] Xu Y.-J., Shi X.-H., Lu J.-H., Qi M., Guo D.-M., Chen L., Wang Y.-Z. (2020). Novel phosphorus-containing imidazolium as hardener for epoxy resin aiming at controllable latent curing behavior and flame retardancy. Compos. B Eng..

[42] Memon H., Liu H., Rashid M.A., Chen L., Jiang Q., Zhang L., Wei Y., Liu W., Qiu Y. (2020). Vanillin-Based Epoxy Vitrimer with High Performance and Closed-Loop Recyclability. Macromolecules.

[43] Liu X., Tian F., Zhao X., Du R., Xu S., Wang Y.-Z. (2021). Multiple functional materials from crushing waste thermosetting resins. Mater. Horiz..

[44] Liu Y., Wang B., Ma S., Xu X., Qiu J., Li Q., Wang S., Lu N., Ye J., Zhu J. (2021). Phosphate-based covalent adaptable networks with recyclability and flame retardancy from bioresources. Eur. Polym. J..

[45] Ma C., Guo Z., Fang Z., Li J. (2022). Flame retardancy and chemical degradation of epoxy containing phenylphosphonate group under mild conditions. Compos. B Eng..

